# Evaluating the Role of Liquid Biopsy to Detect Pathogenic Homologous Recombination Repair (HRR) Gene Alterations in Metastatic Prostate Cancer

**DOI:** 10.3390/cancers17213427

**Published:** 2025-10-25

**Authors:** Soumaya Labidi, Belinda Jiao, Shirley Tam, Parvaneh Fallah, Aida Salehi, Raghu Rajan, Mona Alameldin, Fadi Brimo, William D. Foulkes, Andreas I. Papadakis, Nabodita Kaul, Alan Spatz, Cristiano Ferrario, Ramy R. Saleh, April A. N. Rose

**Affiliations:** 1 Gerald Bronfman Department of Oncology, McGill University, Montreal, QC H3A 0G4, Canada; soumaya.labidi@mail.mcgill.ca (S.L.); belinda.jiao@mail.mcgill.ca (B.J.); shirley.tam@mail.mcgill.ca (S.T.); parvaneh.fallah@mcgill.ca (P.F.); raghu.rajan.med@ssss.gouv.qc.ca (R.R.); alan.spatz@mcgill.ca (A.S.); cristiano.ferrario@mcgill.ca (C.F.); ramy.saleh@mcgill.ca (R.R.S.); 2 Segal Cancer Center, Jewish General Hospital, Montreal, QC H3T 1E2, Canada; aida.salehi@ladydavis.ca (A.S.); andreas.papadakis@ladydavis.ca (A.I.P.); nabodita.kaul.ccomtl@ssss.gouv.qc.ca (N.K.); 3 Lady Davis Institute for Medical Research, Jewish General Hospital, Montreal, QC H3T 1E2, Canada; 4 Cedars Cancer Center, McGill University Health Center, Montreal, QC H4A 3J1, Canada; 5 Department of Pathology, McGill University, Montreal, QC H3T 1E2, Canada; mona.alameldin@mcgill.ca (M.A.); fadi.brimo@mcgill.ca (F.B.); 6 Division of Pathology, Department of Clinical Laboratory Medicine, McGill University Health Center, Montreal, QC H4A 3J1, Canada; 7 Department of Human Genetics, School of Biomedical Sciences, McGill University, Montreal, QC H3A 1Y2, Canada; william.foulkes.med@ssss.gouv.qc.ca

**Keywords:** prostate cancer, HRR, liquid biopsy, ctDNA, somatic, germline

## Abstract

**Simple Summary:**

Metastatic prostate cancers frequently harbour pathogenic aberrations in Homologous Recombination Repair (HRR) genes that confer sensitivity to PARP inhibitors (PARPi). Recent guidelines recommend performing both germline and somatic testing for all metastatic prostate cancer patients, to guide the use of PARPi, and genetic counselling. Tissue is the gold standard for somatic testing. The development of a ctDNA testing alternative is promising as genomic testing of archived tissue leads to a failure rate of up to 30–40% in prostate cancer. We sought to determine whether liquid biopsy combined with tissue testing resulted in a higher rate of detection of HRR gene alterations for patients with metastatic prostate cancer. Dual testing modality (tissue+ctDNA) significantly enhanced the detection rate of HRR alterations 19/58 (32.7%) vs. 29/210 (13.8%) for single testing modality (tissue or ctDNA), *p* = 0.008. The rate of inconclusive results was significantly lower in dual testing modality 0/58 (0%) vs. 25/210 in single testing modality (11.9%), *p* = 0.003. These data highlight a potential role in implementing liquid biopsy—especially in patients who only have older archival tissue available or failed tissue testing—to improve the detection rate of deficient HRR.

**Abstract:**

Background: Metastatic prostate cancers frequently harbour pathogenic aberrations in Homologous Recombination Repair (HRR) genes that confer sensitivity to PARP inhibitors (PARPi). Therefore, accurate identification of all eligible patients is needed. The development of a circulating tumour DNA (ctDNA) testing alternative is promising as genomic testing of archived tissue leads to a failure rate of up to 30–40% in prostate cancer. Methods: This was a bi-institutional retrospective cohort study of patients with metastatic prostate cancer treated at the Jewish General Hospital or the McGill University Health Center, Montreal, Canada, between 2021 and 2023. Molecular data and treatment information were abstracted from a chart review. Chi-square, Fisher’s exact test, and Mann–Whitney tests were used to assess differences between groups. Results: We identified 484 metastatic prostate cancer patients. Somatic and germline testing for HRR was performed in 55.4% (*n* = 268) and 20% (*n* = 97) patients, respectively. Somatic testing was performed on tissue (*n* = 192, 71.6%) or ctDNA from liquid biopsies (*n* = 18, 6.7%) or both (*n* = 58, 21.7%). Pathogenic somatic HRR alterations were detected in 48 patients (17.9%). BRCA2 was the most frequent (*n* = 17), followed by ATM (*n* = 11), then CHEK2 (*n* = 5). Amongst patients with germline testing, 13/97 (13.4%) had pathogenic alterations predicted to lead to deficient HRR, mostly BRCA2 (*n* = 9), and three had detectable BRCA2 in tissue. Dual testing modality (tissue+ctDNA) significantly enhanced the detection rate of HRR alterations 19/58 (32.7%) vs. 29/210 (13.8%) for single testing modality (tissue or ctDNA), *p* = 0.008. The rate of inconclusive results was significantly lower in dual testing modality 0/58 (0%) vs. 25/210 in single testing modality (11.9%), *p* = 0.003. Amongst the 14 patients who had discordant results between liquid and tissue tests, HRR abnormalities were more frequently identified in ctDNA (*n* = 11) vs. tissue (*n* = 3). Patients who had HRR deficiency detected only in ctDNA had older tissue samples (median 5.6 years) compared to those who had deficient HRR detected only in tissue (median 0.2 years; *p* = 0.14). Conclusions: These data highlight a potential role in implementing liquid biopsy—especially in patients who only have older archival tissue available or failed tissue testing—to improve the detection rate of deficient HRR. Our ongoing prospective study will further validate whether the addition of liquid biopsy can identify more patients who are eligible to receive precision therapies by increasing the rate of detection of HRR deficiency compared to routine tissue testing alone.

## 1. Introduction

Prostate cancer is the most commonly diagnosed cancer in males, accounting for about one in five new cancer cases [1]. Despite the high long-term survival in localized prostate cancer, metastatic prostate cancer remains incurable and is the third-leading cause of cancer-related mortality in men [1]. Metastatic prostate cancer (mPC) is a heterogenous disease, and predictive biomarkers are increasingly being used to guide treatment decisions.

Metastatic prostate cancer can either be classified as castration-sensitive (mCSPC) or castration-resistant (mCRPC). In mCSPC, androgen deprivation therapy (ADT) is the standard primary treatment, combined with either androgen receptor pathway inhibitors (ARPI) and/or chemotherapy [2,3,4,5,6,7,8]. Despite optimal treatment, the majority of mCSPC will eventually evolve into mCRPC, for which there is no curative treatment [9].

Deleterious aberrations or alterations in genes involved in Homologous Recombination Repair (HRR) occur frequently in metastatic prostate cancer [10,11,12]. Pathogenic BRCA1, BRCA2, and ATM gene variants are the best characterized and are associated with aggressive disease [10,13,14,15,16]. The PARP enzyme is essential for repairing single-strand DNA breaks. PARP inhibition results in double-strand DNA breaks, which can be repaired via HRR mechanisms. PARP inhibition in cells with loss of function of genes that regulate HRR thus results in excessive DNA damage that cannot be repaired and ultimately causes cell death. As such, HRR gene alterations represent important predictive biomarkers for PARP inhibitors (PARPi) such as olaparib and talazoparib in mCRPC [17,18].

The PROfound phase 3 clinical trial randomized patients with HRR gene alterations to olaparib or physician’s choice in the second-line treatment of mCRPC [19]. Olaparib improved radiographic-progression-free survival (rPFS) and overall survival (OS) [19]. The Talapro-2 trial evaluated the PARPi talazoparib plus enzalutamide vs. enzalutamide alone for the first-line treatment for mCRPC. The combination of talazoparib and enzalutamide was associated with improved rPFS and OS in an unselected population of mCRPC patients [20,21,22]. Combinations of different PARPi with ARPI were also tested in a first-line setting for mCRPC, with statistically significant benefit in rPFS [23,24]. There are ongoing trials testing different PARPi combinations in the mCSPC setting, which, if positive, could change the standard of care treatment for this population of patients [25]. Beyond PARPi, there are emerging data that some HRR alterations (BRCA2 and ATM) may also predict benefit from carboplatin chemotherapy [26].

In Quebec, provincial reimbursement for PARPi for mCRPC is decided based on evidence of predictive biomarkers (BRCA1/2 alterations). The incidence of HRR alterations in prostate cancer data is lacking in Quebec, and little is known about testing practices and challenges in a single-payer health system. In this study, we analyzed data from two academic cancer centres in Montreal, Quebec.

The most recent guidelines recommend performing both germline and somatic testing for all metastatic prostate cancer patients, to guide the use of PARPi, and also for germline to assess other cancer risks and counsel patients’ families [27,28,29]. The data supporting the optimal test type to use (primary tissue, metastasis biopsy tissue, or liquid biopsy) are lacking. However, the ASCO guidelines recommend considering repeat testing either on metastasis tissue biopsy or liquid biopsy for patients with an initially negative test [27,29].

The liquid biopsy concept was introduced for the detection of circulating tumour cells (CTC) over 10 years ago and then extended to circulating tumour DNA (ctDNA) [30,31]. CTC and ctDNA are considered as new biomarkers and subjects of translational research. Clinical applications include early cancer detection, improved cancer staging, early detection of relapse, real-time monitoring of therapeutic efficacy, and detection of therapeutic targets and resistance mechanisms [32].

A key challenge in the management of prostate cancer is accurately identifying all those patients who are eligible for new precision therapies [33]. The development of a ctDNA testing alternative is promising as genomic testing of archived tissue leads to a failure rate of up to 30–40% in prostate cancer. This can be due to low cellularity in fine needle biopsy specimens for pathological analysis, or due to limited quality or quantity of DNA due to degradation over time, or both [33]. Repeating biopsies on metastatic sites, often bone metastases, are invasive, time-consuming, and resource-consuming procedures, which are often futile due to the lack or poor quality of tissue obtained, especially since the decalcification process degrades DNA and leads to NGS failures.

We sought to determine whether liquid biopsy combined with tissue testing resulted in a higher rate of detection of HRR gene alterations for patients with metastatic prostate cancer.

## 2. Patients and Methods

### 2.1. Patient Population, Characteristics, and Outcome

We performed a bi-institutional retrospective cohort study analysis of patients treated for metastatic prostate cancer at two Canadian cancer centres in Montreal, Quebec: Segal Cancer Center, Jewish General Hospital (JGH), and Cedars Cancer Center, McGill University Health Center (MUHC). Patients with proven metastatic prostate cancer with active follow-up in medical oncology clinics between January 2021 and December 2023 were included. Clinical, pathologic, and molecular characteristics were abstracted from chart review. The following characteristics were collected: age, cancer history, pathological findings, stage of disease at the time of diagnosis, site of metastases, and treatment type at localized and metastatic stage. Types of molecular testing, access, time from testing request to results, and detailed results were assessed. We collected the following outcome criteria: investigator-assessed clinical and radiographic response, PSA50 response, time to castration resistance, and OS. Time to castration resistance was calculated from the diagnosis of metastatic disease to castration resistance. OS was calculated from the diagnosis of metastatic disease to death or last follow-up, and from the diagnosis of castration resistance to death or last follow-up. Clinical data were stored in a RedCap database.

### 2.2. Definitions of Molecular Testing Results

HRR alterations were considered when they were reported as pathogenic or likely pathogenic variants. Inconclusive results were defined as either tissue not available for analysis or failure to extract sufficient DNA. The testing methods were consistent across all samples from both institutions. Somatic testing used either an in-house HRR panel or NGS panel, or commercial NGS panels.

### 2.3. Statistical Analysis

Descriptive statistics are provided for patient characteristics. Chi-square, Fisher’s exact test, and Mann–Whitney tests were used to assess differences between groups. The figures were created using the GraphPad Prism 10 (RRID:SCR_002798).

### 2.4. Ethics Approval

Ethics approval for the study was obtained from the Integrated University Health and Social Services Centres (CIUSSS) West Central Montreal REB (Project MP-05-2024-3885) on 12 January 2024.

## 3. Results

### 3.1. Patients’ Characteristics

We identified 484 patients who were treated for mPC in medical oncology clinics between 2021 and 2023. The median age was 67 years (42–92). Most of the patients (*n* = 453, 93.6%) were diagnosed with adenocarcinoma. The Gleason score was ≥8 in 61.6% (*n* = 298) of the cases. More than half of the patients were diagnosed with de novo metastatic disease (*n* = 253, 52.3%). At diagnosis of metastatic disease, 49.2% and 38.8% of the patients had high-volume and high-risk disease, respectively. Median follow-up from the time of diagnosis was 6.6 years, and 3.8 years from the diagnosis of metastatic disease. Patients’ characteristics for the entire cohort are shown in Table 1. We compared the clinical and disease characteristics of patients who had tissue testing vs. ctDNA or tissue and ctDNA testing vs. no somatic testing. We did not observe significant differences between the three groups (Table 2).

### 3.2. Germline Molecular Testing

Germline testing was performed in 20% (*n* = 97) of the cases. Most of the patients who had germline testing also had a personal and/or family history of cancer (*n* = 83), including prostate, breast, and gastro-intestinal cancers (Supplemental Table S1). The majority of the germline testing was performed locally (*n* = 56, 57.7%) through consultation with Medical Genetics. Details are shown in Table 3. The median time for results from the date of testing request by the clinician was 1.6 months (range 0.2–9.7). Pathogenic HRR alterations were identified in 13 patients (13.4%). BRCA2 was the most frequent (*n* = 9, 69.2%), followed by CHEK2 (*n* = 2, 15.4%). One patient had two alterations in BRCA2 and PALB2 genes (Table 3).

### 3.3. Somatic Molecular Testing

Somatic testing was performed for 268/484 (55.4%) patients (Table 4). Somatic testing was principally performed locally at the JGH pathology department (*n* = 193, 72%). The testing was performed by a single modality, either tissue or ctDNA in 71.6% (*n* = 192) and 6.7% (*n* = 18), respectively. Dual modality testing of both tumour tissue and ctDNA was performed for 58 patients (21.7%). Detailed results are shown in Table 4. The median time from testing request by the clinician to the testing results was shorter for ctDNA testing at 0.73 months (range 0.33–4.43) vs. 1.36 months (range 0.06–13.2) for tissue testing (*p* = 0.0006) (Table 5).

**Figure 1:**
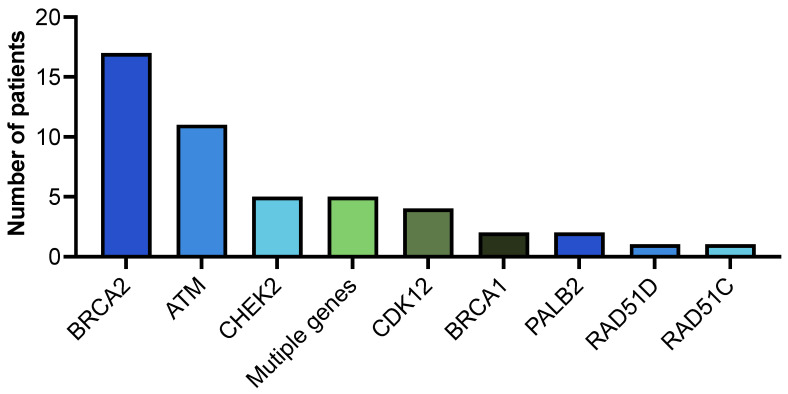
Somatic HRR alterations.

Somatic testing was reported as conclusive in most cases, regardless of the testing modality: 86% for tissue and 88.3% for ctDNA (Table 5). Amongst patients who had somatic testing performed, HRR alterations were identified in 48 (17.9%). BRCA2 alterations occurred most frequently (*n* = 17, 35.4%), followed by ATM (*n* = 11, 22.9%) then CHEK2 (*n* = 5, 10.4%) (Figure 1). Among the 13 patients with identified germline pathogenic HRR alteration, 7 underwent somatic testing. The pathogenic alteration was concordant in both testing modalities in 6/7 (85%) cases: BRCA2 (*n* = 3), CDK12 (*n* = 1), RAD51C (*n* = 1), and BRCA2+PALB2 (*n* = 1).

Somatic HRR alterations were most frequently identified in the BRCA2 gene (*n* = 17), followed by ATM (*n* = 11). Five patients had CHEK2 and multiple gene alterations.

### 3.4. Single vs. Dual Modality Somatic Testing

In this cohort, there were 58 patients who had both tissue and ctDNA analysis. The HRR alteration detection rate was significantly enhanced in these patients (19/58; 32.7%) compared to patients who had only one test—either tissue or ctDNA (29/210; 13.8%; *p* = 0.008) or compared to patients who had only tissue testing (27/192; 14%; *p* = 0.010) (Table 6). The rate of inconclusive results was significantly lower for patients with dual modality testing 0/58 (0%) vs. single modality testing 25/210 (11.9%), *p* = 0.003 (Table 6). Poor DNA quality was the most common reason for an inconclusive testing result (Table 7).

Among the patients who had dual modality testing with both tissue and ctDNA analysis, we observed discordant results in 14 cases (Table 8). Three patients with positive tissue and negative ctDNA were tested at diagnosis of mCSPC, with blood collected 2 to 4 weeks after starting ADT treatment for two of them. Conversely, for 11 patients, tissue results were negative and ctDNA positive. The median age of tissue at the time of testing was 5.6 years compared to 0.2 years in the tissue-positive ctDNA-negative cases.

### 3.5. Treatment with PARPi: Results and Outcomes

Within the entire cohort, 28 patients received a treatment with PARPi. The treatment was received as standard of care in 57.1% of the cases (*n* = 16), mostly in the mCRPC setting (*n* = 26, 92.9%). Detailed results are shown in Table 9. A PSA50 response in mCRPC was obtained in 34.6% of the cases (*n* = 9).

Three patients with HRR alteration detected on ctDNA with negative tissue results were able to receive PARPi treatment as per standard of care, with response achieved in 2/3 cases (Table 10).

## 4. Discussion

Pathogenic HRR alterations occur frequently in prostate cancer. In one study, biallelic inactivation of BRCA2, BRCA1, or ATM was observed in nearly 20% of the affected individuals [11]. In a multicentre study on 692 men with mPC, the incidence of pathogenic germline alterations in the HRR pathway was 11.8% [12]. Mutation frequencies did not differ according to whether a family history of prostate cancer was present or according to age at diagnosis [12]. In our bi-institutional retrospective cohort study analysis of patients treated for mPC, we report an incidence of 13.4% for germline pathogenic HRR alterations, and 17.9% for somatic HRR alterations. BRCA2 was the most frequent in both germline and somatic, 69.2% and 35.4%, respectively.

Genomic alterations in the HRR pathway, and especially BRCA1/2-deficient mPC, show a more aggressive phenotype and poor survival outcomes [13,14,15,16,34]. Apart from the prognostic value, somatic and germline alterations also have a predictive value of response to PARPi and platinum-based chemotherapy, which affects treatment decision-making for patients with mPC [17,18,19,20,23,24,26,34,35,36]. Germline genetic testing may also offer hereditary cancer risk information requiring genetic counselling [12,27,29]. Therefore, the ASCO guidelines [27,29] and the Canadian Urological Association guidelines [28] recommend germline and somatic testing for all mPC. Little is known about the current testing practices in the context of the Canadian healthcare system. In 2022, a cross-sectional survey was conducted by the Canadian Genitourinary Research Consortium (GURC) on an academic multi-disciplinary group of investigators across 22 GURC sites [37]. The results showed that 84% of the investigators were offering genomic testing to patients with advanced prostate cancer, mostly germline (94% compared to 72% on tissue) [37]. Here, we report real-world data for testing practices in two academic centres in Montreal, Quebec. Despite the availability of local tissue testing and genetic consultation, only 55.4% and 20% of the patients were offered somatic and germline testing, respectively. Importantly, provincial reimbursement for olaparib was first approved in Quebec in 2022, which likely influenced local practice patterns.

Numerous guidelines recommend genetic testing in prostate cancer; however, there is a lack of consensus regarding who to test and how the tests should be performed [38,39]. The recommendations used to range from targeted gene test for one or two genes to a prespecified gene panel [38]. The most recently published ASCO guidelines recommend next-generation DNA sequencing panel-based assays (NGS) [27,29]. The NGS-based tests include multiple genes associated with cancer risk factors [40]. The NGS panels are customizable and allow the selection of actionable genes for specific testing purposes such as the HRR pathway genes [39,40]. Tissue is a reliable option and remains the gold standard sample for somatic testing in prostate cancer [39,40]. However, it has some limitations and faces challenges. In fact, the isolation of an evaluable quality and quantity of DNA depends on multiple pre-analytical and analytical factors, such as biopsy route, biopsy technique, tissue processing, and storage of formalin-fixed paraffin-embedded (FFPE) blocks [39,41]. The tumour heterogeneity might result in missing late somatic mutations, especially if testing is conducted on an archival sample [39,42]. There is a higher prevalence of HRR gene alterations in metastatic vs. primary tumour samples [19], but obtaining tissue samples from metastatic sites can be challenging in mPC, as the most frequent site of metastasis is bone, and isolation of DNA from bone requires specific decalcification protocols that may degrade the quality of the DNA [39,43]. In the PROFOUND clinical trial, the rate of tissue test failure was 31% from predominantly archival FFPE tissue samples, with DNA extraction failure as the most frequent cause (13.2%) [19,44]. In our cohort, the rate of inconclusive results on tissue was 12%, with poor DNA quality as the most frequent reason for failure (73.9%). The median age of these samples at the time of analysis was 8 years (2-20). It is proven that the quality of the FFPE samples and the storage conditions can affect the quality of the DNA [44,45,46]. Liquid biopsy and testing on ctDNA offers a valid minimally invasive alternative to tissue testing. Evaluating ctDNA can also provide an overall view of tumour heterogeneity and emerging genomic alterations [32,47,48]. Serial ctDNA testing in mCRPC identifies 11% of new actionable alterations, with 30% of all BRCA2 alterations identified only on repeat testing [48]. The limitations from liquid biopsy include false negatives from low tumour burden and variations according to treatment phase [39,49]. Clonal hematopoiesis of indeterminate potential (CHIP) variants detected on both plasma and whole blood are a known confounder of ctDNA testing [50,51,52]. Paired whole-blood control testing allows CHIP and prostate cancer variants to be distinguished [51]. The ctDNA testing in our study was performed mostly through access programs and clinical trials, with different techniques; therefore, we could not properly assess whether some of the HRR alterations identified in cfDNA were derived from CHIP rather than from prostate cancer ctDNA. This highlights the importance of employing local molecular tumour boards to assist in reviewing and the results from liquid biopsies to help identify such alterations.

We observed a higher HRR gene alteration detection rate in patients who had dual modality testing compared to single modality, and significantly lower inconclusive results. Chi et al. reported an 81% positive percentage agreement and 92% negative percentage agreement for BRCA and ATM status on tissue compared with matched ctDNA samples from the Profound trial cohort [53]. Discordant results with positive BRCA or ATM alteration on tissue and negative ctDNA were found in 19% of the cases [53]. Non-evaluable ctDNA fraction or low ctDNA fractions when evaluable were enriched in these cases, possibly related to low tumour burden [53]. The three cases with discordant results in our cohort were tested in the mCSPC setting shortly after treatment starts, which could lead to low ctDNA fraction. Tissue and ctDNA testing both have limitations and cannot capture all the actionable alterations in all patients, but the availability of both enhances the detection rate of relevant alterations and offers our patients efficient treatment options. We report two cases with negative tissue testing and positive ctDNA serial testing for BRCA2 and BRCA2+PALB2 alterations, who achieved sustained response on PARPi. This illustrates the importance of accurate identification of patients eligible for precision therapies.

## 5. Conclusions

HRR pathway alterations are major prognostic and predictive factors in mPC. Somatic and germline NGS testing is recommended for all mPC to guide treatment planning, but also to guide eventual genetic counselling and cascade testing [27,29]. Tissue testing remains the preferred option; however, it can be challenging in prostate cancer due to limitations from old archival samples, with re-biopsy possibilities especially on bone metastases. ctDNA is a valid alternative, and our data indicate an enhanced detection rate and significantly lower inconclusive results with combining tissue and ctDNA testing. Our study has some limitations, especially the small size and the retrospective character. However, it is a hypothesis-generating study; therefore, we are currently conducting a prospective research project, where all mPC patients will have dual testing modalities, to validate this hypothesis.

## Figures and Tables

**Table 1:** Patients’ characteristics.

*N* = 484		
Centre		*N* (%)
	JGH	240 (49.6)
	MUHC	244 (50.4)
Age Years	Median (Range)	67 (42–92)
Medical History		*N* (%)
Personal history of cancer	Yes	80 (16.5)
	No	396 (81.8)
	Missing data	8 (1.7)
Family history of cancer	Yes	187 (38.6)
	No	173 (35.8)
	Missing data	124 (25.6)
Stage at diagnosis		*N* (%)
	Localized	230 (47.5)
	Metastatic	253 (52.3)
	Missing data	1 (0.2)
Pathology		*N* (%)
Pathology	Adenocarcinoma	453 (93.6)
	Other	3 (0.6)
	Missing data	28 (5.8)
Gleason	7	112 (23.1)
	8	80 (16.6)
	9	189 (39)
	10	29 (6)
	Missing data	74 (15.3)
Treatment for localized disease		*N* (%) *
	Surgery	98 (20.2)
	Radiation	238 (49.2)
	ADT	104 (21.5)
	Adjuvant ARPI (Abiraterone)	5 (1)
	Adjuvant chemotherapy (Docetaxel)	4 (0.8)
	Not applicable	182 (37.6)
	Clinical trial	5 (1)
	Missing data	13 (2.7)
Site of metastases at diagnosis of metastatic disease		*N* (%) **
	Bone	382 (79.5)
	Lymph nodes	194 (40.4)
	Visceral	56 (11.7)
	Other	7 (1.5)
	Missing data	5 (1)
Disease characteristics		*N* (%)
High volume as per CHAARTED trial	Yes	236 (49.2)
	No	211 (43.9)
	Missing data	33 (6.9)
High risk as per LATITUDE trial	Yes	186 (38.8)
	No	225 (46.9)
	Missing data	69 (14.4)
Castration resistance		*N* (%)
	Yes	332 (68.6)
	No	152 (31.4)

* Sums do not add to 100% as one patient may have received multiple treatment modalities. ** Sums do not add to 100% as one patient may have multiple sites of metastases.

**Table 2:** Patients and disease characteristics according to type of testing tissue vs. ctDNA.

Somatic Testing		Tissue Alone *N* = 192	ctDNA or Both *N* = 76	Not Tested *N* = 216	*p*-Value *
Age Years	Median (Range)	67 (42–89)	64.5 (45–82)	68 (46–92)	
Stage at Diagnosis *N* (%)					
	Localized Metastatic NA	83 (43.3) 108 (56.2) 1 (0.5)	33 (43.4) 43 (56.6)	114 (52.8) 102 (47.2)	*p* = 0.124
Disease Characteristics *N* (%)					
High Volume as per CHAARTED trial	Yes	88 (45.9)	37 (48.7)	111 (51.4)	*p* = 0.334
	No	93 (48.4)	28 (36.8)	90 (41.6)	
	NA	11 (5.7)	11 (14.5)	15 (7)	
High Risk as per LATITUDE trial	Yes	71 (37)	31 (40.8)	84 (38.9)	*p* = 0.380
	No	100 (52)	30 (39.5)	95 (44)	
	NA	21 (11)	15 (19.7)	37 (17.1)	
Castration Resistance *N* (%)					
	Yes	146 (76)	61 (80.2)	125 (57.9)	*p* = 0.000
	No	46 (24)	15 (19.8)	91 (42.1)	
Gleason *N* (%)					
	7	46 (24)	13 (17.1)	53 (24.5)	*p* = 0.305
	8	36 (18.7)	10 (13.2)	34 (15.7)	
	9	70 (36.4)	38 (50)	81 (37.5)	
	10	19 (9.9)	3 (3.9)	7 (3.3)	
	NA	21 (11)	12 (15.8)	41 (19)	

* Chi square test.

**Table 3:** Germline molecular testing.

*N* = 484		*N* (%)
Germline testing done	Yes	97 (20)
	No	386 (79.8)
	Missing data	1 (0.2)
Testing access *N* = 97	Local	56 (57.7)
	Trial	20 (20.6)
	Access program	15 (15.5)
	Other	4 (4.1)
	Missing data	2 (2.1)
Pathogenic HRR present *N* = 97	Yes	13 (13.4)
	No	83 (85.6)
	Missing data	1 (1)
HRR alterations *N* = 13	*BRCA2*	9 (69.2)
	*CHEK2*	2 (15.4)
	*RAD51C*	1 (7.7)
	*BRCA2 + PALB2*	1 (7.7)

**Table 4:** Somatic molecular testing access and type.

*N* = 484		*N* (%)
Somatic testing done	Yes	268 (55.4)
	No	216 (44.6)
Testing access *	Local	193 (72)
	Trial	68 (25.4)
	Access program	35 (13)
	Other	5 (1.8)
Testing type	Tissue	192 (71.6)
	ctDNA	18 (6.7)
	Both	58 (21.7)

* Sum does not equal 100% as some patients can have multiple tests.

**Table 5:** Somatic molecular testing: Tissue vs ctDNA.

		Tissue *N* (%)	ctDNA *N* (%)	*p*-Value
Conclusive	Yes	215 (86)	68 (88.3)	0.48
	No	31 (12.4)	7 (9.1)	
	Missing data	4 (1.6)	2 (2.6)	
Time to testing results (months)	Median (range)	1.36 (0.06–13.2)	0.73 (0.33–4.43)	0.0006

**Table 6:** Detection rate and inconclusive results according to testing modality.

	Detection Rate	*p*-Value	Inconclusive Results	*p*-Value
Tissue Alone	27/192 14%	0.55	23/192 12%	0.64
ctDNA Alone	2/18 11.1%		2/18 11.1%	
Single modality: Tissue or ctDNA	29/210 13.8%	0.008	25/210 11.9%	0.003
Dual modality: Tissue and ctDNA	19/58 32.7%		0/58 0%	

**Table 7:** Inconclusive results on somatic testing.

	Reason	*N* (%)	Details
Tissue	Poor DNA quality	17 (73.9)	Median age of tissue at the time of analysis Years (range) 8 (2–20)
	Tissue not available	6 (26.1)	
ctDNA	Undetectable ctDNA	2 (100)	

**Table 8:** Discordant results between tissue and ctDNA.

Patient	Stage at the Time of Analysis	Tissue Conclusive	ctDNA Conclusive	HRR	Tissue Results	ctDNA Results	Tissue Age at Time of Analysis (Years)	Median Age (Years)	*p*-Value
JGH026	mCSPC	Yes	Yes *	*CDK12*	+	-	0.2		0.1236
JGH028	mCSPC	Yes	Yes *	*BRCA1*	+	-	0.1	0.2	
JGH031	mCSPC	Yes	Yes	*PALB2*	+	-	4.0	(0.1–4)	
JGH022	mCRPC	No **	Yes	*CHEK2*	-	+	10.0	5.6 (0.2–12.3)	
JGH029	mCRPC	Yes	Yes	*CDK12*	-	+	2.5		
JGH030	mCSPC	Yes	Yes	*ATM*	-	+	7.0		
JGH032	mCRPC	Yes	Yes	*BRCA2*	-	+	2.5		
JGH033	mCRPC	Yes	Yes	*BRCA2* *PALB2*	-	+	6.0		
JGH034	mCRPC	Yes	Yes	*CHEK2*	-	+	12.3		
JGH035	mCRPC	No **	Yes	*BRCA2*	-	+	11.0		
JGH036	mCRPC	Yes	Yes	*ATM*	-	+	3.3		
JGH164	mCRPC	Yes	Yes	*ATM*	-	+	5.6		
JGH168	mCRPC	Yes	Yes	*CHEK2* *CDK12*	-	+	0.2		
RVH133	mCSPC	Yes	Yes	*ATM*	-	+	0.2		

* Blood for ctDNA testing collected 2 to 4 weeks after starting ADT. ** Poor DNA quality.

**Table 9:** Treatment with PARP inhibitors.

*N* = 28		*N* (%)
HRR alteration	Germline *BRCA2*	2
	Somatic *BRCA2*	9
	Somatic *BRCA1*	2
	Somatic *ATM*	2
	Somatic *CHEK2*	2
	Somatic *CDK12*	1
	Somatic *PALB2*	1
	Somatic *BRCA2+PALB2*	1
	Somatic and germline *BRCA2*	2
	Somatic and germline *BRCA2+CDK12*	1
Type of treatment	Blinded randomized trial	7 (25)
	Open-label trial	5 (17.9)
	Standard of care	16 (57.1)
Stage	mCSPC	2 (7.1)
	mCRPC	26 (92.9)
Line of treatment for mCRPC	First	9 (34.6)
	Second	5 (19.2)
	Third	8 (30.8)
	≥Fourth	4 (15.4)
PSA50 response in mCRPC	Yes	9 (34.6)
	No	13 (50)
	Missing data	4 (15.4)

**Table 10:** Access to PARPi for patients with discordant results between tissue and ctDNA.

Patient	HRR	Tissue Results	ctDNA Results	PARPi Treatment	Stage	Treatment Line	Duration of Treatment	PSA50 Response
JGH032	*BRCA2*	-	+	Olaparib	mCRPC	Fourth Line	3 Months	No
JGH033	*BRCA2* *PALB2*	-	+	Olaparib	mCRPC	Third Line	1.8 years; on-going	Yes
JGH035	*BRCA2*	-	+	Olaparib	mCRPC	Second Line *	3.9 years; on-going	Yes

* Small cell differentiation at progression. PARPi post-etoposide–platinum chemotherapy.

## Data Availability

Primary data are not available for sharing due to patient confidentiality and institutional ethics restrictions.

## Supplementary Materials

The following supporting information can be downloaded at: https://www.mdpi.com/article/10.3390/cancers17213427/s1, Table S1: Germline tested patients: Detailed personal and family history of cancer.
