# Supplementary material for: Evaluating the Role of Liquid Biopsy to Detect Pathogenic Homologous Recombination Repair (HRR) Gene Alterations in Metastatic Prostate Cancer
Source: Cancers (Basel). 2025 Oct 25;17(21):3427. doi: 10.3390/cancers17213427 (PMC12611088; doi:10.3390/cancers17213427)
Supplement: Supplementary file 1 [file cancers-17-03427-s001.zip › cancers-3851160-supplementary.pdf]

Supplemental Table S1: Germline tested patients: Detailed personal and family history of cancer

**Personal history of cancer**	N=19*
Skin, non melanoma	5
Gastro-intestinal	4
Renal	3
Melanoma	2
Neuroendocrine tumors	2
Bladder	2
Hematological malignancies	2
Thyroid	1
Testicular seminoma	1
**Family history of cancer**	N=64**
Prostate	28
Breast	27
Gastro-intestinal	24
Pancreas and cholangiocarcinomas	12
Lung	10
Hematological malignancies	7
Ovarian	5
Gynecological non ovarian	4
Bladder	4
Head and neck	3
Melanoma	1

*Sum does not add to 19 as one patient can have multiple cancers
**Sum does not add to 64 as one patient can have multiple family histories of cancers
